# Coastal Communities, Leisure and Wellbeing: Advancing a Trans-Disciplinary Agenda for Understanding Ocean-Human Relationships in Aotearoa New Zealand

**DOI:** 10.3390/ijerph18020450

**Published:** 2021-01-08

**Authors:** Belinda Wheaton, Jordan Te Aramoana Waiti, Rebecca Olive, Robin Kearns

**Affiliations:** 1 Te Huataki Waiora School of Health, Te Whare Wānanga o Waikato University of Waikato, Hamilton 3216, New Zealand; jordan.waiti@waikato.ac.nz; 2 School of Human Movement & Nutrition Sciences, The University of Queensland, St Lucia 4072, Australia; r.olive@uq.edu.au; 3 School of Environment, Ko te Whare Pūtaiao Faculty of Science, Te Whare Wānanga o Tāmaki Makaurau, University of Auckland, Auckland 1010, New Zealand; r.kearns@auckland.ac.nz

**Keywords:** coastal ecosystems, wellbeing, blue space, surfing, ocean and human health, Mātauranga Maori, Aotearoa New Zealand, transdisciplinary research, coastal recreation, Indigenous knowledges, oceans, local

## Abstract

Commentators are advocating for research to better understand relationships between healthy coastal ecosystems and human wellbeing. Doing so requires inter- and transdisciplinary approaches across humanities, arts, social sciences, and science and technology disciplines. These approaches include culturally diverse knowledge systems, such as indigenous ones, that locate sustainable use of and relationships to marine ecosystems. This paper contributes to this agenda through a case-study of relationships between coastal ecosystems and human wellbeing in Aotearoa New Zealand. This article highlights interconnected cultural and wellbeing benefits of, and socio-ecological relationships between, these coastal ecosystems drawing on a case study of one ocean-based, ‘immersive’ leisure activity, surfing. Further, it examines how these relationships impact human physical, emotional and spiritual wellbeing, and the wellbeing of communities and ecosystems. The research illustrates that surfing creates strong bonds between practitioners and coastal places, linking the health of marine environments and people. We demonstrate the value of a transdisciplinary place-based approach that integrates research across the humanities and social sciences and engages with Indigenous knowledge (Mātauranga Māori). This argument for multicultural co-learning shows the value of Western and Māori vantage points for how we understand coastal blue spaces. Indigenous perspectives, we conclude, deepen appreciation, as well as equity considerations, of how we understand place, wellbeing, and long-term sustainable relationships with marine ecosystems.

## 1. Introduction

Despite increasing global recognition of the ways in which the health of marine environments ‘impacts the health of billions of people’, the relationship between marine ecosystems and human health and wellbeing is still relatively understudied [1]. In recognition of this, the United Nations has declared 2021–2030 the ‘Decade of Ocean Science for Sustainable Development’ [2]. An explicit focus is on achieving the interlinked Sustainable Development Goals (SDG) of ocean conservation and sustainability (SDG14) and human health and wellbeing (SDG3) [3,4]. International research consortiums responding to these interlinked goals have identified a number of key priorities and research gaps [1,5,6,7]. First, there is a need for more multi-, inter- and trans-disciplinary engagement to better understand human-ocean relationships, particularly how the health of marine environments and people are interlinked [1,6,7,8]. Second, this research to date has been dominated by Global-North based research consortiums with a ‘strong European bias in the empirical literature’ [9] (p. 5). This dominance needs expanding to different national contexts including those in the Southern Hemisphere. Third, expansive ecosystems research, and particularly on ‘blue economies’, has highlighted the economic benefits of coastal ecosystems goods and services that provide livelihoods, trade, and food [10,11]. However, this research has not adequately engaged with the different cultural benefits, and ‘dis-benefits’ [12] (p. 487), of these socio-ecological relationships [12,13]. There is an urgent need to focus on how these ‘cultural benefits’ [14,15,16,17] emerge and impact human physical, emotional and spiritual wellbeing, and the wellbeing of communities and ecosystems. Fourth, as philosopher Bradiotti reminds us, while a ‘binary distinction’ between humans and non-human (and nature/culture) has been ‘foundational for European thought since the Enlightenment’, many cultures, including postcolonial and Indigenous epistemologies and cosmologies do not adopt this distinction [18] (p. 2). Indigenous, First Nations, and Global South scholars have long been advocating for Western science to pay better attention to their knowledge systems; that ‘when it comes to human/non-human relations, it is time to start learning from the [Global] South’ [18] (p. 3). While there is widespread recognition that Indigenous knowledge [IK] systems often centralise long-term sustainable use of resources [18,19,20,21,22], IK is marginalised in international research about socio-ecological systems, except for research on/by/for Indigenous communities [20,21,22], and often reinforcing their wider societal marginalisation [22]. The ‘ongoing privileging of one knowledge system’ (‘Western Science’) and suppression of others including Indigenous epistemologies has ‘left Western epistemology so dominant that it can now seem like the only possible framework’ [23]. 

This paper examines the relationship between coastal ecosystems and human wellbeing in Aotearoa New Zealand, focusing on surfing, a popular ‘immersive’ ocean-based recreational activity [24]. (Note: Aotearoa New Zealand is used throughout to signal the nation’s bicultural context, including language English and te reo Māori). This ‘purposive’ [25] case-study addresses these identified research gaps. First, in this island nation in the Southern Hemisphere, people’s relationships with blue-ecosystems from rivers and lakes to estuaries and oceans, provide multiple and overlapping cultural, spiritual, recreational, health, and ecological, as well as economic, values and benefits [26,27,28,29,30,31]. Second, research has shown that surfing can create strong bonds between people and places, shaping participants’ sense of physical, emotional, spiritual, community, social, and cultural wellbeing, impacting people, communities, ‘more-than-humans’ [32] and oceans [33,34,35,36,37,38]. Third, Aotearoa New Zealand’s bi-cultural [23] context, i.e. Indigenous Māori and European settlers (termed Pākehā), provides a revealing cultural context for reassessing the ways in which ‘Western Science’ conceptualises socio-ecological relationships and coastal ‘blue spaces’ as sites of wellbeing [31], that often drive understandings of sustainability, wellbeing, and ocean health. More widely, the paper demonstrates the value of trans- and inter-disciplinary place-based approaches that integrates research across the humanities and social sciences—including cultural and health geography, sociologies of leisure and sport, and feminist cultural studies—and also engages with Indigenous knowledge [IK], specifically Mātauranga Māori.

### Mātauranga Māori, Western Science and Human-Ocean Health and Wellbeing

Before discussing our materials and methods it is important to explain what Mātauranga Māori is, and how this differs from what we will term ‘Western Science’. Western Science is the dominant, and often unquestioned knowledge system in ecosystems (as in most ‘science’ research) underpinned by a ‘Western epistemology’ [23] (p. 83). Mātauranga is a Māori worldview that includes ‘knowledge, wisdom’ and ‘ways of knowing’ [39] (p. 152), a cultural system of knowledge about everything that is important in the lives of the people [23]. Thus, while often treated as ‘merely information’, Mātauranga needs to be understood as a whole knowledge system, epistemology and ontology [23] (p. 83); which ‘includes science’ but also recognises the importance of ‘experiential systems’, emphasising ‘relationship-based learning’ and ‘exploring, theorising and understanding’ at the local level [23] (p. 83). Mātauranga is passed between generations and developed through multiple practices, including arts and technologies [23]. Therefore, as outlined in this paper, Mātauranga Māori has a fundamentally different way of regarding ecosystems to European-based knowledge, implicating how they are lived with and managed [40,41,42,43]. There is no dichotomy between humans and the natural world; rather relationships with the environment include the spiritual, genealogical, affective, cognitive and behavioural ties to a physical location as a result of the meanings and histories within it [40]. 

Although Western knowledge often ‘incorporates knowledge from non-Western epistemologies’ including Indigenous knowledge [IK] systems [23] (p. 86), typically, Western academics treat such IK systems as supplementary to real knowledge, ‘relevant only to the extent that they have something to offer existing theories and discourses’ [44] (p. 72). In other words, the ‘structures of Western science, such as the specific compartmentalisation into disciplines, the hierarchies organising knowledge within those disciplines, and the types of knowledge that are excluded or included’, continue to ‘reflect Western philosophical traditions’ [23] (p. 83). For example, after editing a journal special issue comparing research insights from Mātauranga Māori and Western science, the editor reflected on why there are ‘few explicit references to the Western scientific method as a formalised, culturally specific method of learning’ [45] (p. 164). He observes, ‘the statement that the process of science is “not negotiable” is both misinformed and tending toward bigotry. In fact, Western science has a long history of cultural adaptation and development, and remains open for negotiation of new ways of thinking and learning’. He concludes that, ‘Western culture holds no monopoly on logic-based knowledge or the acquisition of reliable knowledge’ [45] (p. 164).

This paper builds on these insights which, as we show, are central to our transdisciplinary engagement. Furthermore, we recognise that knowledge production is essentially political and historical. Therefore any engagement with Mātauranga Māori perspectives must always recognise past and existing frameworks of power [46]. Mātauranga has been treated as ‘knowledge to be exploited, but not supported’ [23] (p. 84), and Māori have written extensively about the ongoing impact of colonisation on Mātauranga [46], including the ways Aotearoa New Zealand Government ‘policies and systems have marginalised Mātauranga and prioritised Western science’ [23]. While most universities and external research funding agencies now require investigators to show some measure of Māori perspective or commitment to Māori communities in their research, many commentators argue this is a ‘box-ticking exercise’, offering only lip-service to Aotearoa New Zealand being a bi-cultural nation [23,39]. However, initiatives in environmental science are increasingly operating within the Treaty of Waitangi [47] principles, in so doing recognising Māori sovereignty and decision-making power over all things Māori (i.e., environmental resources), and viewing Māori as ‘scientists, experts, collaborators and colleagues’, not simply informants or subjects. For example, Māori researchers in the ‘Our land and water’ National Science Challenge research programme are, ‘combining Mātauranga Māori and Western science’ in ways they suggest ‘will result in a new wave of applied, integrated science that is distinctly designed for Aotearoa’ [48]. We concur that there is not one way of knowing that should shape responses to different local, national and international environmental issues. Our aim in this paper is to show, through engaging in this national context of Aotearoa, that drawing on Indigenous knowledges is ethical and necessary in how we understand, care for, and develop relationships with blue spaces and marine ecosystems.

## 2. Material and Methods

The remainder of this paper is structured as follows: first we outline our transdisciplinary approach to understanding ocean-human health and wellbeing by discussing the different bodies of literature from which this is drawn. Although beyond the scope of this paper, we recognise that transdisciplinary is a term that is used differently across disciplines and subject areas. Here, transdisciplinary refers to knowledge production that is both inter-disciplinary, and also attempts to reflect on and challenge dominant ways of knowing. Engaging in transdisciplinary research creates many challenges, not least the ways in which the ‘knowledge structures, data or mind sets, theories, models, paradigms, norms, values, interests, linguistic forms, and the roles of actors and institutions’ are understood in our different disciplinary approaches [49]. This difficulty of ‘integration’ as Klein [49] outlines, is rooted in long-standing debates in Western philosophy about the assumptions and limitations of ‘different disciplinary’ constructions of the world. However, if transdisciplinary research is to address a societal problem, such as ocean-human health, the emphasis and approach needs to shift away from ‘traditional epistemology’ focused on universality to conflicts of knowledge, hybridity and contextuality’ [49] (p. 11). These important debates are at the heart of the transdisciplinary project. Second, we explain the bicultural context of Aotearoa New Zealand, briefly explaining how Mātauranga Māori influences understandings of coastal environments and the interconnected relationship with human, and ‘more-than-human’, wellbeing [50]. Importantly, we show that this worldview impacts not just those who identify as Māori, but is embedded in a range of informal and formal practices and policies [51,52]. We then discuss existing empirical research on surfing, to show the different, but interconnected, cultural and wellbeing benefits and socio-ecological relationships. This discussion draws on a number of projects the authors have conducted, past and in-process, all of which involve qualitative fieldwork, and in-depth interviews (outlined in Section 2.3). This discussion is supplemented by a new case study of coastal recreation during the first COVID-19 lockdown in Aotearoa conducted April–July 2020, drawing on ‘mainstream’ media, social media, and personal observations (discussed in Section 3). The lockdown responses amongst surfers impacted coastal communities across Aotearoa New Zealand revealed the significance and meanings of coastal recreation generally, and surfing specifically, for the population at large. Finally, we offer some conclusions and recommendations for future work.

### 2.1. Literature Review: Interdisciplinary Approaches to Understanding Human-Ocean Health and Wellbeing

The importance of considering the cultural benefits of the environment to human wellbeing in environmental decision making is increasingly recognised in ecosystems research [14,15,16,17,53] and in social science research on the ‘blue economy’ [54]. For example, researchers exploring the blue economies in Cantabria, Spain argue, while ‘Shipping, fishing, off-shore mining, and energy resources all form crucial links in the value chains of the blue economy’, coastal tourism is one of the most visibly impactful areas of the blue economy and ‘within this sector, surfable waves generate health and happiness’ [10] (p. 65). 

Fish et al. [16] introduced the term ‘cultural ecosystem services’ (CES) to conceptualise the non-material cultural benefits that occur in the interactions between environmental spaces (i.e., physical settings such as rivers, lakes, coasts) and the cultural practices that take place within them. Yet as Bryce et al. [55] outline, there is a dearth of research that explores CES and systematically integrates them into more established ecosystem service assessments. They [55] introduced a typology of relevant cultural practices that includes; playing and exercising; creative and expressing activities; gathering and consuming; and producing and caring. Their research suggests that, through these activities, the multiple cultural wellbeing benefits that develop include place-based identity (e.g., sense of belonging), experiences (e.g., connection to nature), and capabilities (e.g., physical and mental health, skills, and knowledge). Furthermore, through this connection to nature, a sense of belonging and an ethic of care and responsibility can develop which, they suggest, can lead to people supporting the ongoing management and restoration of these ecosystems [55]. This conceptualisation positions CES in a place-based context, recognizing the mutually reinforcing relationships between environmental spaces and cultural practices.

Complementing this research on cultural ecosystem services, and our focus in this discussion, is the more interpretive, place-based and salutogenic research emerging across the social sciences and humanities, and particularly health geography where a so-termed ‘hydrophilic turn’ has emerged [56] (p. 1). In contrast to the dominant pathogenic focus on health risks and problems, this research has explored the factors that support health and wellbeing, particularly the qualities of water which are “affective, life-enhancing, and health-enabling” [56] (p. 2). The authors’ focus on water as healing has a long history across many cultures, with seaside destinations seen as therapeutic sites for leisure and health (e.g., thalassotherapy) [57]. Coastal ‘blue spaces’ have been interpreted as “therapeutic landscapes,” providing physical, psychological, social, and spiritual benefits. These benefits develop from both individual and community experiences and relationships [58] and may arise from immersion or simply visual embrace of the sea [37,58]. This growing body of work has illustrated the different ways in which coastal spaces can foster physical and emotional health and wellbeing across diverse groups, from those who watch, listen, and smell from the shore, to full-immersion activities such as surfing and swimming [56,59,60,61,62,63]. 

Bell et al.’s [61] research in England identified four overlapping “therapeutic experience” (p. 58) dimensions—symbolic, achieving, immersive, and social—in their participants’ local leisure and sport based coastal interactions. From these dimensions, they develop an interdisciplinary model of therapeutic blue space experiences at the coast, which aims to show the breadth and interconnection of different encounters on, by and in the sea. Sharing many of the cultural practices and benefits outlined in Bryce et al. [50], Bell. et al.’s [61] framework recognises that sport and physical activity have an important role in the generation and maintenance of wellbeing, and particularly those activities that involve complete water immersion. As Foley’s research suggests, in such ocean immersions, health and wellbeing is an affective and emotional process [62]. 

Nonetheless, this body of work shows that such relationships are complex and context-specific, and that blue spaces can also bestow risk, trauma, fear, and disengagement impacting human wellbeing and more-than human relationships [31,63]. For example, for those who lack swimming proficiency, beaches can be places of fear and danger, an affective valence potentially exacerbated by the images used in safety campaigns. Issues of safety include increasing waste, which is leading to our lives of “polluted leisure” [64], in which we swim, surf and paddle in the by-products of our consumption [62,63,64,65]. 

Furthermore, some blue-spaces are also sites where individuals and communities can be marginalised or excluded through various economic, political, cultural, and historic processes [31,50,56]. As research across diverse national contexts including England, Ireland, South Africa, USA, and Australia illustrates, coastal spaces can operate as places of white retreat and safety, e.g., [66,67,68,69,70]. At a more local scale, beaches can be ‘colonised’ by activity groups such as surfers, fishers or users of off-road vehicles [71]. Therefore, research needs to also consider the different cultural, economic, socio-demographic, and political factors that contribute to a disconnect with particular coastal bluespaces, as well as those that foster connectedness [35,62]. In contemporary Aotearoa New Zealand, as we show below, it is a contested [71,72] and liminal space where cultural and spiritual significance has been increasingly in friction with the ‘capture’ exerted by real estate and aspirations associated with the capitalist gaze [29]. 

These inequalities and dissonances also relate to how we are able to experience and develop health and wellbeing. Wellbeing is a concept that has been diversely defined across disciplines from neuroscience to social science, therefore recognising these different conceptions of wellbeing is vital [73]. At its heart, wellbeing is about being well, and being implies the ‘whereness’ of living [74]. In contrast to health, which despite recognition of its multidimensional aspects is often more focused on the absence of disease, wellbeing comprises more multidimensional, subjective, and holistic dimensions of a life [74,75,76]. Wellbeing’s complexity lies in the way it connects to a spectrum of social, cultural, economic, spiritual, cognitive, emotional, physiological, and symbolic processes. A useful connection to coastal places can be built around Gesler’s notion of therapeutic landscapes [77], which refers to sites where physical and built environments along with memory and reputation combine to produce an atmosphere conducive to healing. Wellbeing therefore recognises the value of centralising people’s emotions, affects, and experiences [75], as well as the diverse contexts and culturally dependent ways of conceptualizing wellbeing [62]. Within blue space research, ‘place-based’ understandings and promotion of health and wellbeing are increasingly advocated [56], an approach that emphasises the cultural and environmental specificity of wellbeing for specific populations, a perspective that underpins our case study. These concerns are also reflected in Māori models of health and health promotion [78,79,80]. Yet, in settler societies like Aotearoa, the politics of colonisation continue to impact understandings of connections between health, wellbeing and place [71,72,73]. Different contexts affect Indigenous Māori health, and relationships to place amongst Indigenous Māori run deeper and longer than settler-colonizers [81,82]. As Durie [78] (p. 70) suggests, the natural environment is so fundamental to a sense of wellbeing for Māori that tribal elders regard ‘lack of access to tribal lands or territories’ as an important indicator of poor health. Therefore, as Panelli and Tipa [80,83] suggest, a “place-based” approach to wellbeing enables the linking of culture, current and ancestral relationships, and environments into understandings of the interconnections between the health of ecosystems and humans. As we argue in this paper, recognising conceptions of wellbeing, and of place beyond dominant Western, European ideological traditions that have separated humans from ‘nature’, helps to recognise tensions inherent in settler-colonial populations including around blue spaces. Importantly, and while not our focus here, this approach also promotes health leadership by Indigenous peoples whose relationships to colonized places run deeper and longer than settler-colonizers.

### 2.2. Aotearoa New Zealand, Coastal Blue Spaces and Mātauranga Māori 

In Aotearoa New Zealand, the coast has long held an acute significance as a prized source of kaimoana (seafood) pre-colonisation; as a strategic position for pā (villages); as a space of bicultural encounters in early ‘contact’. In the following section, we explain these multiple discourses and practices, and how they connect contemporary Aotearoa New Zealand, the natural world and coast amongst Māori and non-Māori. 

Aotearoa New Zealand’s majestic physical landscape is promoted as a mecca for adventure sports enthusiasts [84]. It has the ‘eighth longest coastline of any nation’ [85] (p. 39) and nowhere is more than approximately 100 km from the sea [85], attracting surfers from around the world, many for extended periods of time. Aotearoa New Zealanders ‘strongly identify with the sea’ and many have ‘lifelong experiences connected with either living close to the sea or visiting for holidays and leisure’ [28] (p. 79) [86,87]. Leisure activities in and on the sea, including swimming, fishing, surfing, sailing, and paddling, play an important role in community life [24,88], providing opportunities for recreational, cultural, ecological and economic (e.g., seafood and livelihoods) activities and benefits [29]. While statistics for informal sports like surfing are often inaccurate [89], national sport participation data show that surfing is a popular recreation along the coastlines of both main islands, year-round [24]. Reflecting international participation trends, the activity is male dominated yet numbers of women participants are increasing, as is inter-generational engagement [89]. 

However, for Māori, who constitute around 67% of Aotearoa’s population, connections to moana (sea) have particular significance [90] having provided physical and spiritual sustenance since the arrival of the seminal voyaging canoes between 800-1350AD [91,92,93]. As we outline in the following discussion, understanding the ways in which Māori develop relationships to coastal blue spaces requires understanding of Māori histories, ontologies and knowledge systems. 

As discussed above, Mātauranga Māori [39] encapsulates a Māori worldview that is underpinned by close relationships between Te Ao Kikokiko (the physical world) and Te Ao Wairua (the spiritual world). A Māori worldview begins with the Creation narrative and Te Kore (the void), an aeon of vast emptiness [91]. Out of Te Kore came Te Pō (a period of darkness), during which the primordial parents Papatūānuku (Earth Mother) and Ranginui (Sky Father) came into being. Papatūānuku and Ranginui were locked in a matrimonial embrace, and during this time a number of their progeny grew tired of being confined to the small and dark space [91]. Tāne-Mahuta (god of the forest) was successful in separating the parents and brought light into the world—Te Ao Marama (the world of light) [91]. From there, the various Māori deities took their respective places within Te Ao Kikokiko and Te Ao Wairua. Whilst slightly different versions exist between some iwi (tribe/s), the creation narrative exemplifies the importance of the environment and the intimate link between humans and natural world. 

Two (of many deities) related to the ocean are Tangaroa (God of the Sea) and Hine Moana (the Ocean Maid). Commonly, Māori refer to the sea and coast in reciting their whakapapa (family lineage) and pēpeha (story of self-identification). A Māori worldview recognises that along with the privileges (food, shelter) associated with the environment, there is also a responsibility to offer care and maintain and sustain it for future generations [40]. Termed kaitiakitanga, practising this ethic ensures that coastal spaces remain a taonga tuku iho (treasures handed down by our ancestors), so that whānau (family) can continue to utilise these spaces for generations to come [91]. Kaitiakitanga ‘weaves together ancestral, environmental, and social threads of identity, purpose, and practice’ [94] (p. 227) and is enacted and manifested in a variety of ways, such as the establishment environmental customary management areas (e.g., rāhui, taiāpure and mātaitai) and paying homage to ancestors and the atua (Māori deities).

Unfortunately, the Mātauranga (traditional Māori knowledge) associated with some of these customs, beliefs and practices is now diminished or lost. The demise of these traditional narratives and customs occurred as a result of British colonisation (e.g., the Tohunga Suppression Act 1907) [95]. The colonisation of Māori and Aotearoa NZ involved the actions of missionaries (to pacify Māori), the military (to keep Māori pacified), and mass immigration (to overpopulate and assimilate Māori). Mass land confiscations uprooted whole communities, sub-tribes, and tribes and decimated Māori trade and economy. Use of te reo Māori (Māori language) was discouraged to the point that it nearly became extinct, much like the Māori population. Despite this, the past 40–50 years has seen a resurgence in Māori identity and self-determination [92]. Part of this resurgence has involved the rise in Māori who are seeking to re-claim this knowledge system to help improve Māori wellbeing and the taiāo (environment). Furthermore, within the sphere of coastal leisure, amongst activities that have seen a resurgence in popularity, are waka ama and waka hourua (voyaging canoes) [96,97]. Alongside an increase in participation numbers within these activities, numerous Indigenous researchers are reclaiming these knowledge systems [98,99].

While internationally surfboard riding has since the 1960s become associated with California [100], surf board riding has long had cultural associations with Indigenous people in Polynesia [35,101]. What is less well known is that heke ngaru (surfboard riding) is also a Māori leisure activity that stretches back to the ancestral home of Māori, Hawaiki, and is also infused with heroic and romantic narratives. While surfing participation in Aotearoa over the recent past has been dominated by Pākehā participants, a small number of Māori researchers have begun to seek out and reclaim the narratives and Mātauranga Māori of heke ngaru (some also use the term eke ngaru e.g., [35]). Although there are periods over time when Māori surfers have not been visible, (and there may have been regional differences in participation levels), Māori have a long history of surfing. Indeed, early British explorers provided the first written accounts of Māori riding waves on various crafts [101,102]. 

Waiti and Awatere [35] found that a sense of place for these kaihekengaru (Māori surfers) related to both the ocean and nearby landmarks. Drawing on the concept of whakapapa, surfing enabled these kaihekengaru to connect with iwi-specific environmental features, their ancestors and the various ātua (Māori deities). Surfing reaffirmed their whakapapa as they are imbued in the environment, immersed in the ocean, and acknowledging the deeds of their ancestors. Much like Rua, Hodgetts, and Stolte [103], the respondents’ ‘sense of belonging is bound to their tūrangawaewae or ancient sense of geographic place(s), as much as it is to their whakapapa’ (or genealogy) (p. 58). In this sense, being Māori is felt, embodied, and emplaced [104] through being, and being Māori, in particular places. Clearly, a Māori worldview, much like their Hawaiian relations [36] embraces distinctive practices and assumptions about what the ocean means and how relationships with it are made.

Contemporary Aotearoa New Zealand, however, is an increasingly multicultural Pacific rim nation with 87.7% of the population now living in an urban environment [85] (p. 33). Immigration and urbanisation have led to increasing hybridity, as well as many more Māori in urban contexts, often becoming disconnected with their Mātauranga. While research suggesting Māori are more likely to report involvement in pro-environment behaviour than Pākehā or those of other ethnicities [85,87], it is also the case that New Zealanders of all ethnicities identify themselves as outdoor people, who have pride in the belief that they live in a ‘cleaner and greener environment than is found in many other developed countries’ [85] (p. 40). Indeed, adopting positive attitudes to the natural environment has been shown to be central to settlement experiences for new migrants for developing a sense of belonging [86]. As Wheaton et al. [31] illustrate, Māori knowledge connected to ‘wellbeing, leisure, and the outdoor world’ continue to have particular significance for Māori but also impact across communities, and to some extent dominant national ‘discourses, policies, and practices’ around coastal leisure activities and outdoor education (pp. 92–93). 

### 2.3. Research Methodology 

In the final section of the paper we explore public responses to the loss of mobility and freedom experienced during the COVID-19 lockdown in Aotearoa with respect to access to beaches and participation in surfing. The initial COVID-19 full-lockdown in Aotearoa New Zealand began 25 March 2020, and continued until 27 April 2020, and was imposed with speed and severity. The first case of the virus was reported on 28 February 2020. At this time public health experts urged the government to act with great urgency, convinced that Aotearoa New Zealand could stop the virus from spreading, and even wipe it out entirely, if a lockdown was implemented strictly and swiftly. The nation returned to Level 1 on midnight 8 June 2020 [105]. These events brought the significance and meanings of coastal recreation generally, and surfing specifically, into sharp relief for Aotearoa New Zealanders. The research team were already engaged with a number of research projects exploring aspects of wellbeing and ocean-based leisure including surfing [30,33,35,89,106,107], which provided some background context. These qualitative research projects involving participant observation (three of the authors are regular surfers) and in-depth interviews with surfers in Aotearoa, including men and women across a range of ages, and self-identified as Māori, Pākehā, and migrants who now call Aotearoa home. Within these projects, all subjects gave their informed consent for inclusion before they participated in the study, in accordance with the Declaration of Helsinki, and as approved by the University of Waikato Human Research Ethics Committee HREC (health) 2018#76 and 2019#06. For this new case study of the COVID-19 lockdown period, (March–July 2020), data was collected via fieldwork (participant observation) and media analysis. All of the research team were living in, or have close connections to, coastal communities during lockdown. Researchers complied field notes of their experiences and daily interactions, including ‘exercise’ walking on the beach, and interactions on social media (i.e., Facebook, Instagram), including sites frequented by surfers locally and around New Zealand. Second, we collated national newspaper articles (between March–July 2020) that discussed beaches, water-sports or surfing. We also included conversations from publicly available social media groups about surfing and water sports (e.g., Surfing New Zealand, Surf Life Saving New Zealand). All social media posts used in the discussion are from public (but unnamed) sources, or permission has been granted. However, for ethical reasons these are all anonymised, and all place names removed. The data—written notes, social media posts, newspaper articles—were analysed thematically [108], and in conversation with theoretical ideas in the literature on coastal immersion in surfing and swimming. 

As discussed above, the integration of different disciplinary paradigm and knowledge systems are at the heart of this transdisciplinary project. Therefore, we briefly highlight the methodological and epistemological assumptions that underpin this research, recognising that for those readers in the ‘natural’ sciences who adopt—at least tacitly—a positivist epistemology, these will not be familiar. Our place-based case-study is rooted in a constructionist interpretivist epistemology, or theory of knowledge. Social-constructionism emphasises that our knowledge and understandings of the world evolve through our active and creative interaction with our environments, other people and other entities. In this paradigm ‘nature’ is not perceived as separate from human culture [108]. Social constructionism also understands the central role of language and communication, which actively constitute or creates these realities which can be multiple and that compete for truth and legitimacy [108]. Such epistemologies often align with inductive methodologies. For example, recognising the contextual ways in which reality is constructed, the research objective necessarily shifts from hypothesis and broad generalisations across society, to reveal the complex and often conflicting ways in which the analysis of peoples’ stories provide insights into understanding experiences [109,110]. These methodologies therefore involve different processes and criteria to natural sciences (informed by a positivist epistemology) impacting all aspects of methodology (i.e., from framing research questions, theory development, through to analysis and the criteria used to present and validate data [110]. This methodology is also complementary with Mātauranga Maori, which, as discussed (in the introduction) also rejects the structures, hierarchies, and types of knowledge embodied in Western philosophical traditions of science. 

## 3. Results and Discussion

### Understanding Ocean-Human Wellbeing: Surfing through Lockdown

The initial COVID-19 full-lockdown brought the significance and meanings of coastal recreation generally, and surfing specifically, into sharp relief for Aotearoa New Zealanders. In the discussion of our data, we exemplify further the themes already shown in the wider literature; the everyday importance of coastal recreation for people’s sense of wellbeing, and specifically surfers; how surfers experience their connection to coastal places; and the ways these connections relate to their individual and collective identities, including forms of belonging and exclusion. 

The unusual circumstances of the lockdown showed how surfers had previously taken-for-granted their mobility and access to the coast. Multiple comments revealed the everyday importance of coastal recreation for people’s sense of wellbeing. This was a typical comment:

‘this is a surreal situation that none of us have ever experienced before. Those of us who live on the coast have never been told we cannot go in the ocean and would never imagine that day would come’.

[111]

During this time of lockdown, debates about the importance of coastal leisure for health and wellbeing, including physical (i.e., exercise), mental, spiritual and economic elements (such as fishing and shellfish gathering) quickly became prominent in national media [112]. Early advice (25 March) from Police Commissioner Mike Bush made it clear that while outdoor exercise was encouraged ‘you are not allowed to drive to the beach or to a park or to anywhere non-essential’ [111]. Surfers interpreted these messages in different ways, with many (along with other ocean-user communities including swimmers, kite- surfers, kayakers, paddlers, stand-up paddle boarders) arguing that they considered surfing as ‘essential’ or as ‘daily exercise’. For example, a man who had been surfing for 50 years, who was interviewed in the national press after receiving a police warning for surfing at his ‘local’ beach [113], regarded being able to go out for a paddle as essential for his (and his wife’s) wellbeing. Unconcerned about the possibility of being arrested, he argued, “[Surfing] is good for our mental sanity” [113]. This sentiment was echoed across the subsequent posts that flooded social media:

‘[surfing] Is essential for mental health purposes’ (surfer, female).

‘policing the ocean is just going to put the New Zealand suicide rate up. Suicide is ALREADY a crisis in New Zealand’ (surfer, male).

‘You can have all the worries in the world and they instantly vanish when you go for a surf. Other people de-stress in different ways but it seems to me that surfers are a lot more connected to their medium—but then, I’m talking from my selfish perspective.’

[114]

The experience of being denied access to the ocean spaces they are usually able to surf unimpeded, offered insight into the degree to which surfers rely on oceans and beaches for their health and wellbeing. The perspectives expressed above offer a “salutogenic” way of thinking about human health and the ocean, in that they are focused on the ocean as health-giving and health-enabling [115]. Adapting Gesler’s [77] idea introduced earlier, these surfers experience oceans and beaches as a “therapeutic seascape” seen as essential to their health and wellbeing. Yet, like the surfer who risked arrest due to the importance of the beach he feels to his mental health [113], social media posts suggest surfers had never thought of this experience as anything less than an entitlement which caused further tensions. 

In response to broader restrictions on movements, more Aotearoa New Zealanders than usual visited beaches, including for surfing during the initial period of lockdown [113]. As with coastal towns internationally, the high numbers of people visiting beaches near coastal communities where they were not residents, caused tension both amongst community members, as well as against non-residents:

‘If some tourists was [sic] to try and drop in or snake my wave, I tell them don’t do it again or I'll cough on you’ (social media)

Some individuals in the surfing community, such as within local board rider clubs, tried to mobilise collective responses in their communities [116]. For example,

‘We understand the pent-up frustration and anxiety that will build up over this time if you cannot get out in the water. But this is about the bigger picture of what we as a world are facing. This is about our communities and the lives at risk. We are fortunate to live in this place and we are lucky to call it home. We need to do everything in our power to protect’ (social media).

‘If one person goes surfing, one more will go, then one more, and more. The more crowded the lineup, the more dangerous the lineup’ (social media. Note: The ‘line up’ is the term used by surfers to describe where they sit in the water to catch the waves. It can often be quite busy).

By Saturday 4th April 2020 a joint release from the Ministry of Health and New Zealand Police stated that ‘New Government rules specifically prohibit surfing and boating,’ and swimming [117]. They reasoned that these activities ‘could lead to the participant requiring medical treatment or search and rescue efforts’ [104]. This rationale was linked to the potential pressure on existing services should COVID cases have risen suddenly, and the limited health services in rural areas. As an earlier social media post from Surf Life Saving New Zealand [118] explained:

Please NO rock fishing, fishing from a boat, surfing, kite surfing, knee boarding, stand up paddle boarding, paddling, snorkelling, surf ski, or diving etc etc—during the Covid-19 lockdown. ... and yep we know it sucks. If you get into trouble, you take our emergency services away from where they need to be. … STAY HOME SAVE LIVES.

This complete ban on surfing, and limited access to the beach including for collecting kaimoana (seafood) and fishing, led to vociferous debate, which continued across all media and featured prominently amongst coastal communities including on social media (i.e., community Facebook pages). Posts publicly ‘called out’ those who had surfed. This policing of surfers and water users impacted many residents in coastal towns and cities. Some surfers were strident in their opposition—some to the lockdown rules, and others to those individuals or the authorities who flaunted them [111,113,119,120,121].

‘The guy was clearly asking ‘what on earth are you doing during the lockdown’ but [the surfer] was clearly not bothered about that and then gave him the bird.‘

[120]

Despite government rhetoric of ‘our team of 5 million’ being in this together and ‘being kind’ to each other [105], on-line ‘vigilantism’ particularly through community-based social media, was prevalent as some surfers continued to ignore the rules, while also speaking out against the public and police who took them to task [111,115,119,120]. Coastal communities and surfers particularly were under surveillance, from the police, and from each other. The following online ‘conversation’ was typical on community social media:

Person 1 Bloody idiots, surfing (place name)—I can see several people via the [web]cam... come on stay home. If you mess this up we’re all going to be grounded for longer. Selfish. [39 comments, 40 likes and some dislikes]
Person 2 Someone go down and pop all the tyres on their cars, wankers.
Person 3 Someone call the cops [8 likes]
Person 4 Go for a freaking surf [9 likes]

Lockdown exposed and amplified some of the underlying tensions in these communities, such as between surfers and non-surfers, ‘locals’ [68], and non-locals (variously defined), and those who are permanent house dwellers and so-called ‘freedom campers.’[51] The latter group, characterised on social media as ‘foreigners living in vans,’ were often accused of leaving their rubbish, and ‘unlike kiwi surfers’ had ‘no respect for the coastline’ (social media posts). The sense of ‘them and us’ between the coastal community and city dwellers, was highlighted in multiple posts, such as a lockdown ditty critiquing people coming to their holiday homes (attributed to Abby Lawrence), which was re-posted by a surfer on a community social media page and received over 100 ‘likes’. In some cases, these claims to being local’ and disputes about going surfing were also tied to whakapapa (genealogy), such as ‘If your bloodline ties to [place] you’re an original local. By all means come and stop me’ (social media post).

Amongst some Māori a further tension existed with public mistrust and a sense of needing to keep themselves safe. Road closures in the Far North region were established by local iwi (tribal) leaders to protect their communities from the pandemic [121,122]. Māori recalled their experiences from the 1919 Spanish influenza epidemic that greatly affected those communities [123] and were therefore concerned that people would come from the cities (i.e., COVID hotspots) back to their ‘baches’ (Note: this is a term for holiday or beach houses) and bring the virus into these small rural communities. Indeed, the road closures were a pro-active public health response that promoted tribal mana whakahaere (autonomy). Social media posts from the general public including some Māori showed a general lack of disregard and disrespect towards this public health measure. These posts often cited personal grievances against one of the tribal leaders, or commonly referred to the narrative of Māori blocking access to the beaches, a widespread, yet incorrect, narrative perpetuated by those who think Māori have and continue to deny access to beaches [124,125]. 

During this time, another roadblock was established on an access road to a Northland beach by local iwi, to stop the potential spread of COVID [125,126]. The fear was that if surfers accessed the beach, then others would do the same. In this case, however, locals (both Māori and non-Māori) seemed supportive of this measure, with the only backlash appearing to come from international tourists [122]. Moreover, a similar roadblock had occurred at this same place in mid-2019 at this same place in response to the vandalism of culturally established pou (carved wooden post) which demarcated a shellfish collecting exclusion zone. This customary rāhui (ban) has been established to ensure that the local shellfish populations are protected and are able to rejuvenate [124]. During the lockdown, a similar rāhui was established on the Waipa and Waikato rivers (by the Māori king, Kiingi Tuheitia) [126]. This ban prohibited food gathering and all recreational activities on the waterways. This ban was established to ensure the mauri (life-force) of these waterways were able to recover and to contain the spread of COVID. This was explained as being “about informing the people and letting everyone know that, actually, because the waterways are a source of spiritual inspiration for our people, they need time to recover too”. This action exemplified the pro-active response of Māori throughout Aotearoa New Zealand to protect both their community, and the environment.

However, these narratives also speak to the sociality of surfing and how surfers of all ethnicities are deeply embedded in the economic and cultural wellbeing of coastal communities, as noted here:

‘I’ve got mates who have been mates with each other for 30 years and they’ve fallen out over whether we should be surfing. It’s brought the worst out in people because they can’t do what they love’

[114].

As the following social media post on a community group about lockdown anxiety illustrates, coastal communities worked hard to restore a sense of wellbeing and belonging:

‘Our community strengths include our commitment to our people and our environment, the care we have for each other and our ability to collaborate and work together. Well done everyone! Let’s keep caring for each other, continue being courageous and compassionate, continue to stay at home and save lives—We are all in this together’ (social media).

Later, when Aotearoa New Zealand moved from full lockdown (Level 4) to Level 3 (13 May 2020), one of the first changes to attract national headlines was the reinstating of right to surf and fish [127]. Despite government advice to ‘stay local’ and that it was ‘not a time to try something new’, it was not just regular surfers who returned to Aotearoa New Zealand’s beaches en masse [128,129]. Rather, around the country surf breaks had much higher than usual numbers of people, including those who had not been in the water for many years, as well as first-time surfers. A Wellington surfer of 28 years reported [130] ‘he had never seen so many surfers there at once’ and that ‘It was like a seal colony of 150 plus surfers stretching across the whole bay’ in contrast to up to 40 people on a regular weekend. News reports confirmed that Kiwis took to the beach in their masses, ‘in the water, walking their dogs, watching the sunrise and all sorts’ [128]. This beach-going and surfing trend was still apparent several months later, and through winter. As one surf shop owner observed, ‘I can’t remember ever selling so many wetsuits; we just can’t get enough’ (field notes).

While the COVID-induced lockdown circumstances were exceptional, they help amplify and highlight the deeply affective ‘everyday’ experiences of recreational surfers’ practices and how they influence people’s sense of wellbeing, collective identities, and forms of belonging and exclusion [33,34]. This unusual time-period has emphasised the socio-cultural relationships, experiences, and meanings that surfers bestow on their experiences of oceanic blue spaces, in diverse local and national contexts [33,34,63]. 

Of course, it is not only surfers who experience these connections—Foley also describes swimming as a ‘healing activity’ [62] (p. 218)—but in recent scholarship surfing has been recognised as fostering particularly strong connections. As research on ‘surf therapy’ wellbeing initiatives that have been proliferating around the world [30,130,131] has also shown, these wellbeing benefits of ocean immersion are multi-sensory. lisahunter and Stoodley [34] explored surfers’ relationships with salt-water, using various technologies to capture their multi-sensory experiences, feelings and ‘entanglements with water’ (p. 4). Their work focused less on what participants thought about surfing, but rather on their experiences of ‘seeing, hearing, and feeling it’, such as ‘the smells and sounds of the coast, the temperature and movement of the water, and the taste of salt in the air’ (p. 8). Their research [34] helps explain how the saltwater ecosystems in which surfers are immersed are central to the health-enabling aspects of surfing as an ocean practice. They also show how the benefits of surfing are not only in the water itself, but also the connection with human and non-human others in the water [34]. For example, participants explained the pleasures of ‘waiting for a wave, the splashing of the water, and sociality with others, both friends or casual acquaintances’ (p. 19). They also referred to ‘how the absence of surfing made them feel frustrated or unhappy’ [34] (p. 12). Nonetheless, the ‘buzz’ of surfing can endure beyond the surf session. For many, the opportunity to go surfing is infrequent, yet simply recalling the memory of the experience, for some sustained wellbeing benefits [129]:

‘I could still “feel” a buzzing, an openness, and a noticeably big smile that swept over me, creating a second moment of wellbeing’.

[34] (p. 11)

These experiences and relationships were consistent with those described during lockdown, such as one surfer who said, he would ‘surf every day if he could’, but during lockdown has ‘been visualising surfing to compensate’. Others simply watched the ocean, feeling mounting frustration at being unable to get into the water:

‘Scott, who lives in Gisborne, has seen some of New Zealand’s best surfers sitting and watching the waves this week, frustrated that they can’t surf. The surf has been ideal. A barrelling wave at Wainui that Scott caught on camera from dry land, with police blocking access to the beach’.

[114]

In summary, the all-consuming nature of surfing was often expressed in the social media posts around COVID lockdowns, showing the ocean’s place as a therapeutic landscape and surfing’s importance for peoples’ perceived wellbeing.

The lockdown situation was also informing in understanding the often-contradictory nature of individual’s relationship with the natural world. Some surfers posted photos of the unusually empty beaches, landscapes, and seascapes with comments reminding their social media friends of the majesty of their surrounds, and the joy and privilege these nature-scapes could bring such as ‘watching a sunset’. Such comments reflect the literature showing New Zealander’s strong identification with the coast [28], positive attitudes to the natural environment, which creates a sense of belonging [67,87]:

‘on the West Coast of NZ watching the sun. Feeling the arms of safety take over...... Enjoy your safety’ (male surfer).

‘I feel lucky. My lockdown experience has been one of neither isolation or loneliness, … Just a few minutes of intentional quiet each day, whether sitting or perhaps walking in nature, with practice, is all it takes to feel steady’ (female surfer).

Others discussed the joys of less human impact (e.g., few cars, airplanes overhead), and the different sounds (e.g., ‘hearing the birds not traffic’). For example, ‘Back to Nature, Stayin Positive. Stoked to see my feathered friend today.’ Another example was a sunset photo titled ‘Day 7 Level 2’:

… ‘you can hear the Oyster Catchers churping or communicating to each other, and then a group fly off for their nighttime location of which I do not know? The Oyster catcher vocals are one of my fav. Stay Positive. Enjoy settled weather’ (social media).

However, discussion of the health and wellbeing of the places themselves, while evident, was less visible. Social media posts from Pākehā and Māori referred to the benefits of removing humans from landscapes and seascapes, seeing this as a time for the earth to ‘heal’ and for the balance to be ‘restored’ [131]. For example, recognising the problems of recreational and commercial fishing for local ecosystems, one social media comment read: ‘The sea looks so peaceful at the moment, I wonder if the fish would replenish after couple of weeks of lockdown. What are the chances of dolphins making a return?’ Indeed, a traditional Māori world view would view the lockdown period as providing a chance for Papatuānuku and Tangaroa to heal and re-align from human pressures, as this post exemplified:

‘Papatuanuku, Ranginui & Tongaroa are sighing a breath of relief. They are regenerating and resetting the earth and ocean’

As explained Papatūānuku and Tangaroa are central to the Māori creation narrative that exemplifies the intimate link between humans and natural world. This healing of te taiāo (the environment) was exemplified when a school of kanae (mullet—a type of fish) was seen along the unusually clear shorelines of Okahu Bay in central Auckland. Some of the local kaumātua of Ngāti Whātua (one of the tribes located in central Auckland) recalled that they hadn’t seen kanae there since before they were ‘booted up the hill’ following the land confiscations in 1951 (social media post). Often Māori (surfers and non-surfers) also pointed out that the ocean was not solely for surfing; that the beach was ‘like a pātaka kai (pantry) where people actually go gathering for food around the shoreline’, or a place to engage with the Māori deities (social media post). As food gathering was prohibited in lockdown, many iwi (tribes) sent care packages to tribal members, no matter where in the country they were. Some of the more remote iwi also incorporated local delicacies (i.e., seafood and other wild foods) in the packages. However, broader discussion about whether and how sea/landscapes were impacted by the absence of humans, who are usually a part of these ecosystems, was not prevalent amongst surfers. For example, was there perhaps more plastic left uncollected after being washed up on beaches, or animals who missed their regular human companions? To be clear, we are not advocating for a version of environmental sustainability that excludes humans, yet despite surfer’s common assertions of themselves as environmental stewards [33,132] and of the role ocean places play in their health, there was less reflection amongst surfers on such possibilities. Furthermore, many surfers were only able to see what they were being denied, rather than seeking out alternative nature space environments such as the forests or hills, which as research has shown also have therapeutic benefits [61]. The lack of water access caused a level of anxiety amongst surfers that did not translate to. In this sense, surfers’ relationships are, perhaps, place-myopic rather than ‘place-responsive’ [133].

Our intention here is not to suggest, in a comparative sense, that this lockdown was particularly hard for surfers or coastal communities. Rather we have suggested that this outpouring of frustration, anxiety and anger was because of the loss of a practice so embedded in daily life that its impact on people’s physical and spiritual wellbeing was previously taken for granted. However, as previous research has shown, blue spaces can also be sites of exclusion [33,56]. In this case study, the surfer’s language and attitudes were highly localised and exclusive; many did not believe that beach access should be extended to all individuals. Indeed, reflecting previous research, some surfers saw themselves as having deeper oceanic relationships than other citizens [132], and therefore, they claimed greater ‘rights’—even ‘needs’—to access their ‘local’ beaches than more casual water users. The impact of these beliefs was the exclusion of some individuals and groups, and therefore that these wellbeing benefits are not available and extended to all.

Our case study contributes to the growing body of work illustrating the different ways in which coastal spaces are therapeutic landscapes that can foster physical and emotional health and wellbeing from those on the shore, to full-immersion activities such as surfing [56,59,60,61,62]. It also shows the value of ‘place-based’ understandings and the promotion of wellbeing [56,133], which emphasises the cultural and environmental specificity of wellbeing for specific populations. While lockdown had many impacts for people’s wellbeing including economic influences, such as loss of jobs, income from tourism, and access to food. These were not dominant in these surfers’ stories. Therefore, as Fish et. al. [16] and other in CES research have argued, research needs to reveal these important ‘non-material cultural benefits that occur in the interactions between environmental spaces (i.e., coasts) and the cultural practices (i.e., surfing) that take place within them.

## 4. Conclusions

This paper contains advanced thinking around the need for more transdisciplinary engagement to better understand ocean-human relationships. In particular, our concern has been for how the health of marine environments and people are interlinked, and how Indigenous perspectives can deepen appreciation, as well as equity considerations, of understandings of place and the performance of wellbeing. We have acknowledged the ways that Indigenous knowledge systems centrally locate the ontological significance and long-term sustainable use of marine resources. The contradiction, however, is that despite aspirations for sustainable seas and coastlines, such knowledge is persistently marginalised in international research concerning these socio-ecological systems. The exception is research on, by, and for Indigenous communities which, in remaining an important yet niche endeavour, continues to reinforce a position of Indigenous marginalisation in society and the academy and policy sphere. As researchers, not only do we need to explore the knowledges relevant to the places, spaces and communities we research, but also recognise that Indigenous peoples can offer place-specific leadership that cares for place while also challenging oppressive histories. 

Our paper has attempted to bring to international scholarship an argument for transdisciplinary research via ‘bicultural co-learning’ through an interweaving of Western and Māori vantage points on the significance of marine blue spaces through the example of the ocean-based ‘immersive’ leisure activity surfing. Our research demonstrated that the activity is more than a recreational pursuit on and in the ocean’s surface layer. Rather, it is a metaphorical as well as literal (and near-littoral) immersive practice creating strong bonds between surfers and coastal places. While Māori and Pākehā surfers to some extent continue to embody different knowledges and meanings, these bonds were experienced across these surfing communities. 

We have demonstrated the value of a transdisciplinary, place-based approach that integrates inter-disciplinary research across the humanities and social sciences, including cultural and health geography, sociologies of leisure and sport, and Indigenous knowledge, in this case, Mātauranga Māori. Despite its pervasiveness in the everyday experience in Aotearoa New Zealand, the relationships between marine ecosystems, Mātauranga Māori and human health and wellbeing are still relatively under-studied. There is emerging evidence to suggest that the processes used in Māori ‘science’ have an important role in removing the boundaries between disciplines and helping teams of scientists with different areas of expertise to integrate their knowledge [22,41,42,47]. These processes are not only inspiring ‘new conceptions of knowledge and approaches to creating knowledge’, but are also helping to develop the ‘skills that promote conversation and learning from different knowledge systems’ [41] (p. 86). In other words, they enable an ecological way of thinking about wellbeing such that, in Aotearoa, a partnering between Western and Māori science is allowing an incorporation of Māori wellbeing ontologies. As researchers develop more integrated and transdisciplinary work on human and ocean health and wellbeing, we encourage researchers to engage with the Indigenous knowledge systems that reflect the values of the places they are working with and from. In so doing, transdisciplinary research teams can develop more integrated methods to identify practical, ethical, and sustainable solutions. 

## Data Availability

N/A Data is contained within the article or supplementary material as referenced.
